# Septate Junction Proteins Play Essential Roles in Morphogenesis Throughout Embryonic Development in *Drosophila*

**DOI:** 10.1534/g3.116.031427

**Published:** 2016-06-03

**Authors:** Sonia Hall, Robert E. Ward

**Affiliations:** Department of Molecular Biosciences, University of Kansas, Lawrence, Kansas 66045

**Keywords:** septate junction, morphogenesis, head involution, dorsal closure, salivary glands

## Abstract

The septate junction (SJ) is the occluding junction found in the ectodermal epithelia of invertebrate organisms, and is essential to maintain chemically distinct compartments in epithelial organs, to provide the blood–brain barrier in the nervous system, and to provide an important line of defense against invading pathogens. More than 20 genes have been identified to function in the establishment or maintenance of SJs in *Drosophila melanogaster*. Numerous studies have demonstrated the cell biological function of these proteins in establishing the occluding junction, whereas very few studies have examined further developmental roles for them. Here we examined embryos with mutations in nine different core SJ genes and found that all nine result in defects in embryonic development as early as germ band retraction, with the most penetrant defect observed in head involution. SJ genes are also required for cell shape changes and cell rearrangements that drive the elongation of the salivary gland during midembryogenesis. Interestingly, these developmental events occur at a time prior to the formation of the occluding junction, when SJ proteins localize along the lateral membrane and have not yet coalesced into the region of the SJ. Together, these observations reveal an underappreciated role for a large group of SJ genes in essential developmental events during embryogenesis, and suggest that the function of these proteins in facilitating cell shape changes and rearrangements is independent of their role in the occluding junction.

Pleated septate junctions (hereafter referred to as SJs) are found in all ectodermally derived epithelia (*e.g*., epidermis, salivary glands, hindguts, and tracheae) in invertebrates, where they localize basal to the adherens junction (Noirot-Timothee *et al.* 1978), and function to prevent paracellular flow between the apical and basal sides of an epithelium, much as tight junctions provide a barrier function in vertebrate epithelia (Urakabe *et al.* 1970; Lord and DiBona 1976). More than 20 genes have been implicated in the establishment or maintenance of SJs in *Drosophila* (reviewed in Izumi and Furuse 2014). Many of these genes encode membrane proteins with extracellular motifs suggesting a role in cell adhesion. Early in development (stage 12 of embryogenesis, or about 8 hr into the 24 hr embryonic period) most SJ proteins are membrane associated and line the length of the lateral domain. In stage 13 embryos, some SJ protein can be observed in intracellular puncta that colocalize with early and recycling endosomal markers, while the majority of the protein remains localized to the lateral membrane (Tiklova *et al.* 2010). In stages 14 and 15 SJ proteins are gradually enriched at the apical lateral region, although considerable protein can still be detected along the lateral membrane. At stage 16 (about 14 hr after egg laying), SJ proteins are finally tightly localized to the apical lateral region that defines the SJ. Electron microscopic studies revealed the presence of dispersed electron-dense intercellular septae in wild-type embryos beginning at stage 14, and increasing in number and regularity until an ultrastructurally mature SJ is established in stage 17 (Tepass and Hartenstein 1994). Functional studies revealed that the paracellular barrier is not established until late-stage 15 in wild-type embryos (Paul *et al.* 2003). The correct organization and function of epithelial SJs requires that each member of the complex is present, suggesting that the SJ is a large, stable, and highly cross-linked protein complex (*e.g.*, Ward *et al.* 1998; Genova and Fehon 2003).

Mutations that result in the loss of SJs in embryonic epithelia and glia are embryonic lethal, with paralysis due to the loss of the occluding function at the blood–brain barrier in glia (Baumgartner *et al.* 1996). Most of the studies characterizing SJ genes note this embryonic lethality, but focus on the cell biological role of the SJ protein in the organization and function of the occluding junction. A few studies, however, have characterized defects in developmental events associated with these mutations. For example, we initially identified *Macroglobulin complement-related* (*Mcr*) in a screen for genes involved in imaginal disc morphogenesis during metamorphosis (Ward *et al.* 2003; Hall *et al.* 2014). Additionally, zygotic loss of function alleles of *coracle* (*cor*) and *Neurexin-IV* (*Nrx-IV*), as well as maternal/zygotic mutations in *Discs large* (*Dlg*), result in defective dorsal closure (DC) during stages 13–14 of embryogenesis (Perrimon 1988; Fehon *et al.* 1994; Baumgartner *et al.* 1996). Furthermore, loss of *Fasciclin III* (*FasIII*) results in defective hindgut morphogenesis that normally occurs in stage 13 of embryogenesis (Wells *et al.* 2013). Finally, mutations in many SJ genes were initially identified as having highly convoluted embryonic trachea, suggesting a requirement for SJ genes in tracheal morphogenesis (*e.g.*, Behr *et al.* 2003; Wu *et al.* 2004, 2007; Batz *et al.* 2014).

Here we set out to determine whether an essential role in embryonic morphogenesis is a common function of all the SJ proteins, or if it rather reflects a pleiotropic effect of a small number of proteins that also happen to function in the establishment or maintenance of SJs. To address this question we undertook a reevaluation of mutations in nine core SJ genes for their role in embryonic morphogenesis. We chose alleles that had been previously characterized for their cell biological role in the SJ, and found that all nine are essential for numerous developmental events during embryogenesis, suggesting that at least a large collection of SJ proteins participates in morphogenetic events shaping the body plan and organs in *Drosophila*. Detailed examination of salivary gland (SG) organogenesis reveals that SJ proteins are required for both cell rearrangements and cell shape changes that drive the morphogenesis of this tissue. Interestingly, the timing of the developmental events that are perturbed by loss of function SJ mutations precedes the biogenesis of the occluding junction, suggesting distinct roles for these proteins in morphogenesis and in forming the SJ.

## Materials and Methods

### Drosophila stocks

All *Drosophila* stocks were maintained on media consisting of corn meal, sugar, yeast, and agar in a room that typically fluctuated between 21.5° and 23°. Genetic experiments were conducted in incubators controlled at a constant temperature of 25°. The following SJ mutant strains were used: *Cont^ex956^* (Faivre-Sarrailh *et al.* 2004), *cor^4^* (Ward *et al.* 1998), *cor^5^* (Ward *et al.* 2001), *kune^c309^* (Nelson *et al.* 2010), *Lac^G00044^* (Llimargas *et al.* 2004), *Mcr^1^* and *Mcr^EY07421^* (Hall *et al.* 2014), *Nrg^14^* (Hall and Bieber 1997), *Nrg^17^* (Paul *et al.* 2003), *nrv2^ZCL1649^* (Buszczak *et al.* 2007; Hall *et al.* 2014), *Nrx-IV^4304^* (Baumgartner *et al.* 1996), and *Tsf2^KG01571^* (Tiklova *et al.* 2010). *w^1118^*, *actin (act)-Gal4*, *daughterless (da)-Gal4*, *Df(3R)BSC146*, *Df(2R)BSC696*, *Df(2R)BSC305*, *cor^5^*, *FasIII^E25^*, *kune^c309^*, *Lac^G00044^*, *Nrg^14^*, *Nrg^17^*, *nrv2^ZCL1649^*, *Nrx-IV^4304^*, and *Tsf2^KG01571^* were obtained from the Bloomington *Drosophila* Stock Center (BDSC, Bloomington, IN). *UAS-kun-RNAi* (stock 3962), *UAS-Mcr-RNAi* (stock 100197), and *UAS-Nrg-RNAi* (stock 107991) strains were obtained from the Vienna *Drosophila* RNAi Center (VDRC, Vienna, Austria; Dietzl *et al.* 2007) . The *Nrg-GFP* Fly Trap line G00305 (Morin *et al.* 2001) and the *Nrx-IV-GFP* fly trap line CA06597 (Buszczak *et al.* 2007) were obtained from the FlyTrap consortium (Yale University School of Medicine, New Haven, CT). *fkh-GAL4*, *UAS-GFP* (Wang *et al.* 2008) was obtained from Arash Bashirullah (University of Wisconsin, Madison, WI). *Cont^ex956^* was obtained from Manzoor Bhat (University of Texas Health Science Center, San Antonio, TX). *cor^4^*, *Mcr^1^*, *Mcr^EY07421^*, *nrv2^ZCL1649^*, and *act-Gal4* were balanced with *CyO*, *P{w^+^*, *Dfd-EYFP}*, *Nrg^14^* and *Nrg^17^* were balanced with *FM7c*, *P{w^+^*, *Dfd-EYFP}*, and *Cont^ex956^*, *Kun^c309^*, *Lac^G00044^*, *Nrx-IV^4304^*, and *Tsf2^KG01571^* were balanced with *TM6B*, *P{w^+^*, *Dfd-EYFP}* to allow for unambiguous identification of embryos (Le *et al.* 2006).

### Cuticle analysis

**Figure 1:**
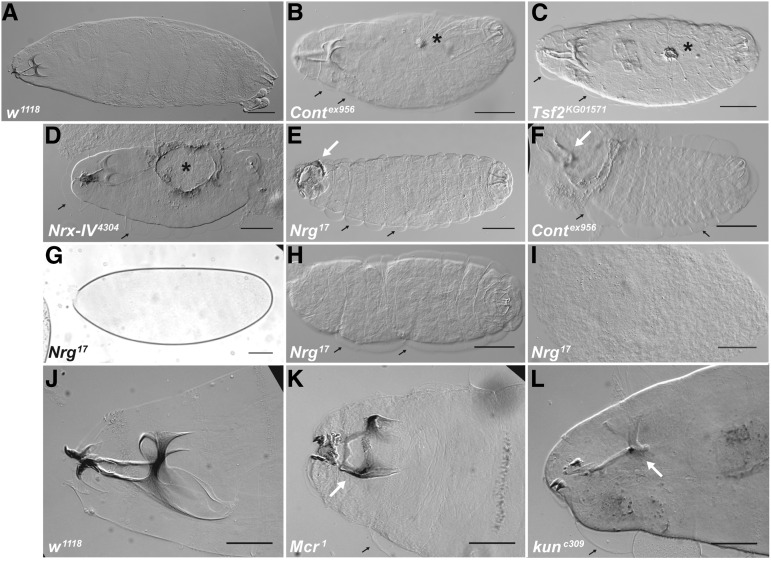
Loss of function mutations in SJ genes result in embryonic lethality with defects in head involution and dorsal closure. DIC photomicrographs (A–F, H–L) and a brightfield photomicrograph (G) of cuticle preparations of *w^1118^* (A and J), *Cont^ex956^* (B and F), *Tsf2^KG01571^* (C), *Nrx-IV^4304^* (D), *Nrg^17^* (E and G–I), *Mcr^1^* (K), and *kune^c309^* (L). Anterior is to the left and dorsal is up or facing. Defects in dorsal closure are indicated by cuticular scabs or holes on the dorsal surface (asterisks in B–D), whereas defects in head involution vary from completely uninvoluted head structures (arrows in E and F), to incomplete head involution where head skeletal structures are compressed anteriorly (arrow in K), to underdeveloped head skeleton (arrow in L points to a head skeleton lacking most of the dorsal and ventral processes). A number of embryos showed an “Empty Cuticle” phenotype (G), that when devitellinized varied from having a thin cuticle barely capable of holding the embryo together (H), to little or no cuticle resulting in disorganized mass of cells (I). Note the cuticle has separated from the epidermis in all of the mutant animals (small black arrows), but not in the *w^1118^* animals. Scale bars = 100 μm in A–F, H, and I, 60 μm in G, and 50 μm in J–L.

Embryos from SJ mutant strains and deficiencies balanced with *FM6*, *P{w^+^*, *Dfd-EYFP}*, *CyO*, *P{w^+^*, *Dfd-EYFP}*, or *TM6B*, *P{w^+^*, *Dfd-EYFP}* were collected on apple juice agar plates for 1 hr at 25°, aged for 17 hr and then selected based upon the absence of Yellow Fluorescent Protein (YFP). Nonhatched embryos 48 hr after egg laying were dechorionated in 6% sodium hypochloride, mounted in Hoyer’s medium on microscope slides, and cleared for 24 hr at 50°. All cuticular phenotypes were scored on a Nikon Eclipse 80*i* compound microscope. For the devitellinized cuticles shown in Figure 1, mutant nonhatched embryos 48 hr after egg laying were dechorionated in 6% sodium hypochloride, devitellinized by shaking in a 1:1 mixture of methanol and heptane, expanded by heating at 37° in 1X phosphate buffered saline plus 0.1% Triton X-100, mounted in CMCP-10 (Polysciences, Inc, Warrington, PA), and cleared overnight at 50° on microscope slides. The cuticles were imaged using differential interference contrast microscopy (DIC) on a Nikon Eclipse 80*i* compound microscope equipped with a Photometrics CoolSNAP *ES* high-performance digital CCD camera. The *w^1118^* control cuticle was from a first instar larva < 30 min after hatching that was mounted in Hoyer’s medium on a microscope slide, and cleared for 24 hr at 50°. Photomicrographs of the cuticles were cropped, rotated, and adjusted for brightness and contrast with ImageJ (Schneider *et al.* 2012), and figures were compiled in Adobe Illustrator (version CS6, San Jose, CA). All experiments were performed in triplicate and means with standard deviations were determined. Statistical significance was calculated using a Fisher’s exact test.

### Immunostaining

**Figure 2:**
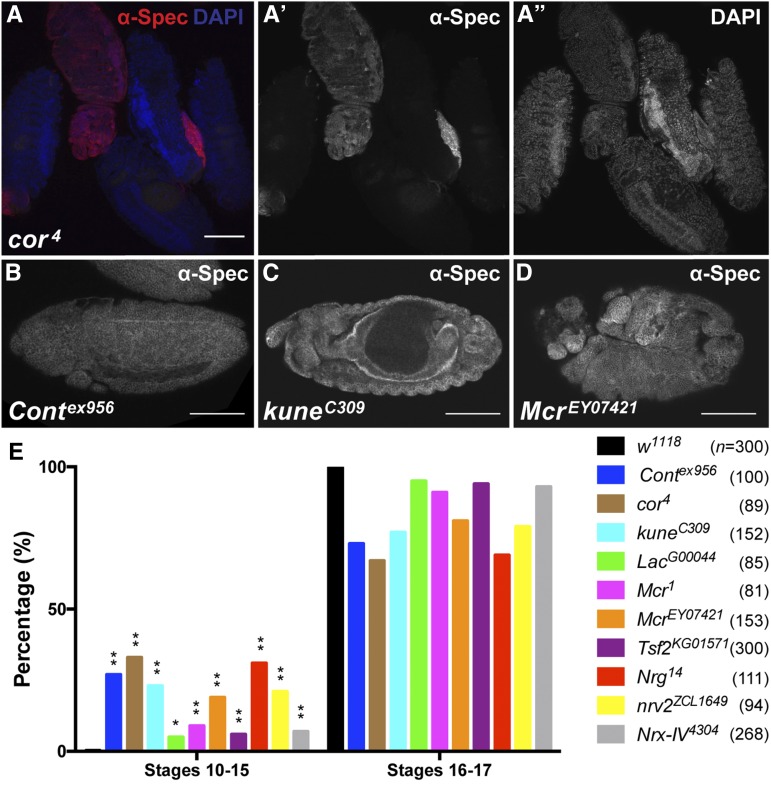
Mutations in SJ genes result in early arrested embryos. (A) Confocal optical section of a field of embryos 17–18 hr after egg laying from a self-cross of *cor^4^/CyO*, *Dfd-YFP* adults stained with antibodies against α-Spectrin (red and in channel A’) and with DAPI (blue and in channel A”). Note that four of the embryos have secreted cuticle and are refractory to antibody staining, whereas the second embryo from the left is a *cor^4^* homozygous mutant (identified by absence of YFP, not shown) that has not secreted cuticle and can be stained. (B–D) Confocal optical sections of *Cont^ex956^* (B), *kune^c309^* (C), and *Mcr^EY07421^* (D) mutant embryos aged 17–18 hr after egg laying and stained with antibodies against α-Spectrin to show the general morphology of the embryo. Anterior is to the left. Note that the *Cont* embryo has arrested prior to the initiation of germ band retraction, the *kune* embryo is arrested at late stage 14/early stage 15, and the *Mcr* mutant embryo is generally disorganized. (E) Quantitation of developmental stages of *w^1118^* and SJ mutant embryos from a 1 hr embryo collection, subsequently aged 17 hr and then stained with antibodies against α-Spectrin. Note that all 10 SJ mutants had a significantly higher percentage of embryos arrested prior to stage 15 than the *w^1118^* control (Fisher’s exact test). *n*, total numbers of embryos scored for each genotype. * *P* < 0.05, ** *P* < 0.0001. Scale bars = 100 μm.

Embryos were fixed and processed for antibody staining as described in Fehon *et al.* (1991). Detailed protocols are available at https://www.protocols.io/view/Immunohistochemistry-Drosophila-Embryo-cutwwm. Embryos were collected for 1–2 hr or overnight depending on the experiment and aged to the appropriate developmental stage as determined by gut and head morphology. Antibody concentrations for all the primary antibodies used in this study can be found at protocols.io at https://www.protocols.io/view/Optimized-Concentrations-for-Developmental-Studies-daq2dv?guidelines. Antibodies against α-Spectrin, ATPα, Coracle, and DE-Cadherin were obtained from the Developmental Studies Hybridoma Bank (University of Iowa, Iowa City, IA), whereas antibodies against Nrx-IV and Contactin were obtained from Mazoor Bhat (University of Texas Health Science Center, San Antonio, TX), antibodies against Nrg were obtained from Nipam Patel (University of California, Berkeley, CA), and antibodies against Kune were obtained from Mikio Furuse (Kobe University School of Medicine, Kobe, Japan). FITC-labeled antiphosphotyrosine antibodies were obtained from Sigma-Aldrich (St. Louis, MO) and used at 1:800. Secondary antibodies (Jackson ImmunoResearch Laboratories, West Grove, PA) were used at 1:800. Confocal images were acquired on an Olympus FV1000 confocal microscope (Olympus America, Inc., Center Valley, PA) equipped with Fluoview software, a Zeiss LSM510 Meta Laser Scanning confocal microscope (Carl Zeiss Inc, Thornwood, NY), or a Zeiss LSM700 AxioImager.Z2 confocal microscope. Photomicrographs were cropped, rotated, and adjusted for brightness and contrast in ImageJ. Figures were compiled in Adobe Illustrator (version CS6). The statistical significance for the experiment to address early arrested development (Figure 2) was calculated using a Fisher’s exact test.

### Morphometric analysis and nuclear quantification of SGs

Confocal z-series stacks were collected on wild-type and SJ mutant embryos mounted such that lateral views of SGs could be imaged immediately under the epidermis using a Zeiss LSM700 AxioImager.Z2 with a Plan-APOCHROMAT 63X/1.4 Oil DIC lens using a step size of 0.39 μm. Individual images were stacked in Image J to generate a 3-dimensional reconstruction. We used these reconstructions to generate cross-sectional views at three positions roughly equally spaced along the long axis of the gland, and did this for 10 glands per genotype. The number of SG cells per cross-section was determined by counting the number of Hoescht-stained nuclei in three independent cross-sections per gland as described (Chung and Andrew 2014). We also measured the length of two lateral membranes per cell and the intervening apical membrane for a total 10 cells per gland. Statistical significance was calculated using a Student’s *t*-test.

### Data availability

*Drosophila* strains and antibodies generated in our laboratory (anti-Mcr and anti-Uif) are available upon request. Raw confocal z-series stacks used to generate cross-sectional views used in the morphogenetic analyses described in Figure 5, Table 2, and Table 3 are available upon request.

## Results

### Loss of zygotic expression of SJ genes results in head involution and dorsal closure defects

We initially identified mutations in *Mcr* in a screen for genes required for imaginal disc morphogenesis during metamorphosis (Ward *et al.* 2003; Hall *et al.* 2014). Coupled with previous studies showing a requirement for the core SJ genes *cor* and *Nrx-IV* during dorsal closure (DC) (Fehon *et al.* 1994; Baumgartner *et al.* 1996), we wondered whether other SJ genes were required for morphogenesis, and for which developmental events they were required. In this way we could address whether an essential role in morphogenesis was a general feature of SJ gene products, or whether a few of these genes have pleiotropic effects that included this function. To address this question, we conducted terminal phenotypic analysis on animals with zygotic loss of function mutations in nine core SJ genes that have been well characterized in the literature to result in defects in SJ organization and function. We first examined the cuticles of mutant animals that failed to crawl away 48 hr after egg laying. The cuticle is secreted late during embryogenesis, and can be used to assess the major developmental events shaping the body plan including germ band retraction, head involution, and dorsal closure. The most obvious defect observed for all of these mutations was an inability to complete head involution (Figure 1). We noted a large variation in the expressivity of this phenotype, from a complete inability to invert the head segments (Figure 1, E and F) to minor defects in head skeleton positioning and morphology (Figure 1, K and L). The penetrance of head involution defects in embryos that secreted a cuticle varied from about 46% of *cont^ex956^/Df(3R)BSC146* to 100% of *Tsf^KG01571^* embryos (Table 1). Using the same set of cuticle preparations, we observed similarly variable defects in DC. These phenotypes ranged from puckering of the dorsal epidermis and small cuticular scabs (Figure 1B), to small (Figure 1C) and large dorsal holes (Figure 1D). DC phenotypes were less penetrant than head involution defects, but were nonetheless present at some level in every line examined (Table 1). By observing cuticles from devitellinized embryos, we also noticed that the cuticle had separated from the epidermis in most of the mutant animals examined (small black arrows in Figure 1), but had not done so in the *w^1118^* control animals (Figure 1, A and J). This phenotype had been previously reported in *cor* mutant embryos (Lamb *et al.* 1998), but not for other SJ mutants. Also consistent with the previous report about *cor* mutant phenotypes, we observed fainter denticle belts (that were nevertheless correctly patterned), and deposits in the areas of the SGs in nearly every mutant cuticle examined (data not shown).

**Table 1:** Cuticle phenotypes of SJ mutant embryos

Genotype	% Empty Cuticle Phenotype*^a^* (*n*)*^b^*	% Head Involution Defective*^c^*	% Dorsal Closure Defective*^c^*
*Cont^ex956^*	22.4 ± 8.6 (245)	68.7 ± 27.1	5.9 ± 1.4
*Df(3R)BSC146 / Cont^ex956^*	21.3 ± 13.8 (337)	46.2 ± 14.6	6.2 ± 1.9
*cor^4^*	53.5 ± 4.5 (314)	80.5 ± 17.3	27.6 ± 2.9
*kune^c309^*	50.7 ± 4.5 (417)	87.2 ± 12.8	5.1 ± 5.7
*Df(2R)BSC696 / kune^c309^*	31.4 ± 8.0 (358)	55.8 ± 20.0	6.3 ± 2.6
*Lac^G00044^*	61.5 ± 12.0 (356)	81.4 ± 25.4	2.6 ± 2.5
*Df(2R)BSC305 / Lac^G00044^*	42.9 ± 10.7 (358)	78.1 ± 5.1	5.5 ± 2.7
*Mcr^1^*	47.3 ± 19.3 (394)	97.5 ± 4.2	19.5 ± 11.2
*Mcr^EY07421^*	28.9 ± 6.5 (467)	74.7 ± 15.9	1.1 ± 1.2
*Nrg^17^*	64.6 ± 26.8 (490)	99.6 ± 0.6	1.6 ± 1.4
*Act5c > Nrg-RNAi*	73.3 ± 33.0 (408)	72.6 ± 15.1	0.9 ± 1.3
*nrv2^ZCL1649^*	26.4 ± 16.9 (201)	96.9 ± 6.3	3.0 ± 2.1
*Nrx-IV^4304^*	25.4 ± 16.1 (474)	95.8 ± 3.8	41.4 ± 16.9
*Tsf2^KG01571^*	26.2 ± 27.3 (336)	100 ± 0	5.2 ± 3.6

a Mean plus standard deviation from at least three independent experiments.

b Total number of embryos examined in all experiments.

c Mean plus standard deviation of all the embryos that produced a cuticle from all the experiments.

To verify that these observed phenotypes were due to the loss of the SJ genes and not due to second site mutations on the mutant chromosomes, we examined animals hemizygous for a subset of these mutations, as well as animals ubiquitously expressing RNAi against SJ genes, and observed similar defects in DC and head involution (Table 1). These observations demonstrate that it is the zygotic loss of SJ gene function that is responsible for defective head involution and DC, thus indicating an essential role for multiple SJ genes in these morphogenetic developmental events.

During the analysis of cuticle phenotypes in the SJ mutants we observed a number of embryos that did not produce any observable cuticle (Figure 1G). This phenotype varied from ∼20% to 65% in different SJ mutant lines, and was even higher for ubiquitous expression of *Nrg-RNAi* (Table 1). When these embryos are devitellinized they vary from having a thin cuticle that has some recognizable features (Figure 1H), to essentially no cuticle, resulting in a disorganized tissue mass (Figure 1I). To characterize developmental defects associated with these embryos, we collected embryos from a 1 hr egg lay, aged them for 17 hr, and stained them with antibodies against α-Spectrin and with 4′,6-diamidino-2-phenylindole (DAPI). At this stage, wild-type animals have secreted a cuticle that prevents their examination by indirect immunofluorescence, whereas many mutant animals (identified by a lack of Yellow Fluorescent Protein expressed from balancer chromosomes) could be examined in this way (Figure 2A). Notably, all 10 of the SJ mutant alleles we tested had a significantly higher percentage of embryos arrested prior to stage 16 (as judged by gut morphology and degree of head involution) than *w^1118^* control embryos (Fisher’s exact test; Figure 2E). Examples of arrested mutant embryos are shown in Figure 2, B–D, demonstrating a range of terminal phenotypes from those arrested as germ band extended embryos (Figure 2B), to ones showing severely disorganized body plans (Figure 2D).

### SJ proteins are expressed in early embryos coincident with major morphogenetic events

**Figure 3:**
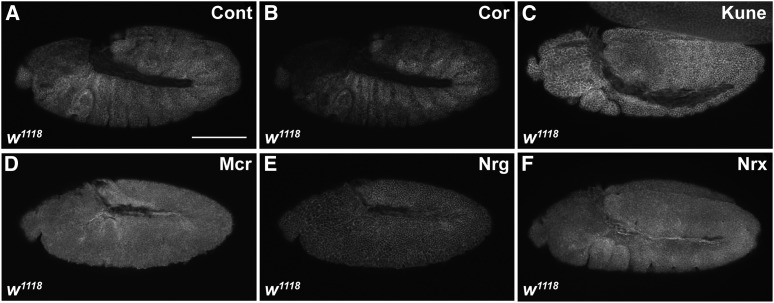
SJ proteins are expressed as early as stage 10 of embryogenesis. Confocal optical sections of stage 10–11 *w^1118^* embryos stained with antibodies against Cont (A), Cor (B), Kune (C), Mcr (D), Nrg (E), and Nrx-IV (F). Scale bar = 100 μm.

The finding that SJ genes are required for morphogenetic events that happen as early as germ band retraction raised questions about the protein expression of these genes during early stages of development. Typically, studies of SJ genes have monitored the expression of these proteins beginning in stage 12–13 embryos where they localize all along the lateral membrane prior to assembling into the region of the SJ beginning in stage 15 (*e.g.*, Tiklova *et al.* 2010). In order to gain a better understanding of their expression prior to these stages, we used immunohistochemistry to examine the expression and subcellular localization of eight core SJ proteins. These experiments revealed that all eight SJ proteins (Cor, Cont, Kune, Mcr, Mtf, Nrg, Nrv, and Nrx-IV) are expressed as early as stage 10 of embryogenesis where they are associated with the membrane (Figure 3 and data not shown).

### Mutations in SJ genes show defective morphogenesis of embryonic SGs

**Figure 4:**
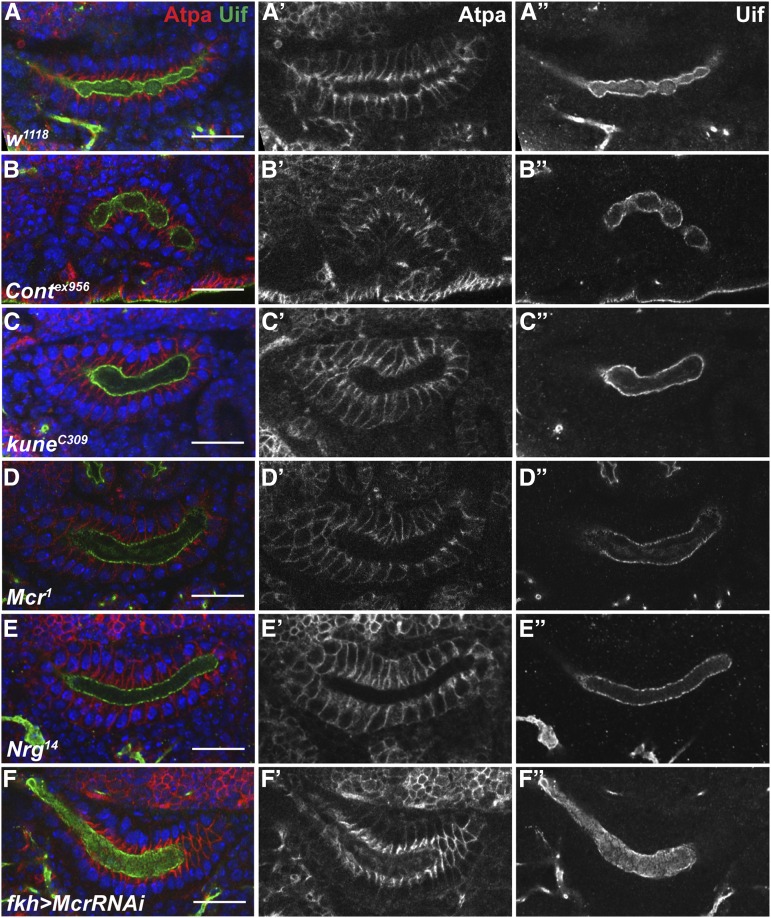
SJ mutant SGs exhibit abnormal morphology. Single confocal optical section selected from z-stack that revealed the largest number of cells in cross-section from stage 16 *w^1118^* (A) *Cont^ex956^* (B), *kun^c309^* (C), *Mcr^1^* (D), *Nrg^14^* (E), and *fkh-GAL4 > Mcr-RNAi* (F) SGs stained with antibodies against ATPα (red, and in individual channels in center panels) to outline cells and against Uif (green, and in individual channels at far right) to mark the apical membrane, and with DAPI (blue) to mark the nuclei. Note that many of the SJ mutant SGs are shorter and fatter than *w^1118^*, and have a broad lumen. The *Nrg^14^* SG (E) is most similar to wild type. Scale bars = 20 μm.

Given our results showing that SJ genes are required for morphogenetic events during midembryogenesis, we set out to examine if these genes are also required for morphogenetic processes during organogenesis that occur at the same time. The salivary gland is an epithelial tube that begins to form at stage 10 when cells invaginate from the ventral surface (Myat *et al.* 2005). The SG elongates through a process of coordinated cell shape changes and collective cell migration where the distal tip cells extend protrusions and drive the migration of the gland over the visceral mesoderm (Bradley *et al.* 2003; Vining *et al.* 2005). Since the SG reaches its full extension by stage 15 of embryogenesis, we examined SGs (immunostained with antibodies against ATPα and Uif) from stage 16 animals mutant for *Cont^ex956^*, *kune^C309^*, *Mcr^1^*, and *Nrg^14^* (Figure 4). In all cases the SGs invaginated and migrated, giving rise to organs that could be unambiguously identified as SGs. Nevertheless, the SGs from each mutant line exhibited abnormal morphology characterized by short and fat glands. In *kune^C309^* (Figure 4C) and *Mcr^1^* (Figure 4D) mutant animals, the most notable phenotype was an abnormal broad morphology of the lumen of the SG. In addition to abnormal lumen morphology, *Cont^ex956^*, *Mcr^1^*, and *kune^C309^* glands often displayed a bent appearance suggestive of aberrant migration. Of these, *Cont^ex956^* glands are the most abnormal, often folding back upon themselves (Figure 4B). We knocked down *Mcr* and *Kune* expression specifically in the SG using RNAi driven by *fkh-GAL4*, and observed identical phenotypes to those produced in the loss of function alleles (Figure 4F and data not shown). These results indicate that the SG defects in the loss of function SJ mutations are not due to second site mutations, and further suggest that the requirement for these genes in morphogenesis is tissue autonomous.

### SGs from SJ mutant embryos exhibit defective cellular rearrangements and cell shape changes

**Figure 5:**
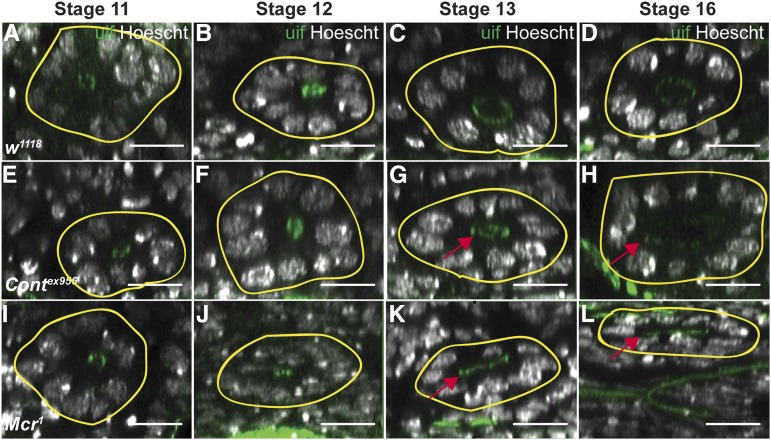
Developmental time course of SG organogenesis from wild-type, *Cont^ex956^*, and *Mcr^1^* mutant embryos reveals defective cell rearrangements in the mutant glands. Confocal z-series rendered in xz transverse cross-section of stage 11–16 *w^1118^* (A–D), *Cont^ex956^* (E–H), and *Mcr^1^* (I–L) SGs stained with antibodies against Uif to mark the apical membrane and Hoescht to label nuclei. The basal surface of each SG is indicated by the yellow line. Note that the number of nuclei surrounding the lumen in *w^1118^* SGs decreases from stage 11 to stage 16, whereas the number of nuclei surrounding the lumen of SJ glands does not. These views also highlight the broad, unexpanded or asymmetric lumen found in the mutant glands (arrows in G, H, K and L). Scale bars = 100 pixels.

To examine the cellular defects associated with the aberrant SG phenotypes in SJ mutant animals, we fixed and stained *w^1118^*, *Cont^ex956^*, *Mcr^1^*, *Nrg^14^*, *kune^C309^*, *fkh-GAL4 > Mcr-RNAi*, and *fkh-GAL4 > kune-RNAi* stage 16 embryos with Hoescht and antibodies against Uninflatable (Uif; Zhang and Ward 2009) and ATPα, and collected confocal z-series from 10 SGs from each collection. We made 3-dimensional reconstructions of these confocal z-series in ImageJ, and generated cross-sectional views at three positions roughly equally spaced along the long axis of the glands. We quantified the number of nuclei surrounding the lumen at each of the three positions in all 10 glands for the SJ mutants and *w^1118^*, and determined that there were more nuclei (and therefore cells) surrounding the lumen of SJ mutant glands than in wild-type glands (Figure 5 and Table 2). For a subset of SGs from each line, we quantified the total number of nuclei per gland and found that there were no differences between *w^1118^* and any of the SJ mutant lines examined (data not shown). We then conducted similar analyses on SG from *w^1118^*, *Cont^ex956^*, and *Mcr^1^* mutant glands from embryos staged between 11 and 13 (Figure 5 and Table 2). In wild-type embryos there are on average 10.6 cells surrounding the lumen of the gland at stage 11, whereas by stage 16 that number decreases to 7.5, consistent with cell rearrangements that have been reported earlier (Xu *et al.* 2011). Stage 11 and 12 *Mcr^1^* and *Cont^ex956^* SGs had similar numbers of cells surrounding the lumen to wild-type animals, suggesting that the initial formation of the gland was largely normal. The number of nuclei in cross-section at 16, however, was not significantly different from the number of cells surrounding the lumen at stage 11, but was significantly different from the number of cells in stage 16 *w^1118^* glands, indicating that these mutant glands either failed to undergo cell rearrangements, or were strongly reduced in their rearrangements relative to wild type (Table 2).

**Table 2:** Cell number surrounding the lumen of wild-type and SJ mutant SG glands*^a^*

Genotype	Average Number of Nuclei Stage 11*^b^*	Average Number of Nuclei Stage 12*^b^*	Average Number of Nuclei Stage 13*^b^*	Average Number of Nuclei Stage 16*^c^*
*w^1118^*	10.6 ± 0	9.8 ± 1.0	8.2 ± 0.2	7.5 ± 0.3
*Cont^ex956^*	10.4 ± 1.0	10.4 ± 0.5	10.6 ± 0.8**	9.9 ± 0.9***
*Mcr^]^*	10.3 ± 0.7*^d^	11.3 ± 1.2	10.8 ± 1.1*	9.2 ± 1.1***
*kune^c309^*	ND	ND	4.3 ± 0.5	10.1 ± 1.6***
*Nrg^14^*	ND	ND	4.2 ± 0.7	8.4 ± 1.3*
*Fkh > Mcr-RNAi*	ND	ND	3.8 ± 0.5	9.0 ± 1.2**
*Fkh > Kune-RNAi*	ND	ND	3.3 ± 0.3	9.4 ± 0.6***

* *P* < 0.05, ** *P* < 0.001, *** *P* < 0.0001. ND, not determined.

a Cell number is inferred by the number of Hoescht-stained nuclei.

b Mean plus standard deviation of nuclei number surrounding lumen of SG at three evenly spaced positions along the proximal distal axis of the gland. *n* = 3 different glands.

c Mean plus standard deviation of nuclei number surrounding lumen of SG at three evenly spaced positions along the proximal distal axis of the gland. *n* = 10 different glands.

d Statistics indicate difference between SJ mutant and *w^1118^* at the same stage of development.

The analysis of z-series reconstructions also allowed us to examine the lumen morphology that we noted to be aberrant in the SJ mutant stage 16 glands (Figure 4). Interestingly, the broad lumen phenotype frequently observed in SJ mutant glands was not due to an overall increase in lumen diameter, but rather was associated with a substantial asymmetry of the lumen that often appeared flat or collapsed (Figure 5L). In addition to the flat lumen phenotype, we observed variations in diameter along the length of the lumen from very narrow to wide, resulting in a convoluted appearance that bends and turns through focal planes (data not shown), explaining the cystic-like lumen phenotype observed in the *Cont^ex956^* mutant by single confocal section of a lateral view (Figure 4B). Interestingly, we did not observe any noticeable variation in lumen diameter in *Nrg^14^* mutants through these z-series reconstructions (data not shown), consistent with what we observed by single confocal section (Figure 4E).

Defective apical membrane expansion along the P/D axis of the developing gland has been shown to contribute to defects in lumen width (Pirraglia *et al.* 2010). In addition, Maruyama and Andrew demonstrated that changes in apical membrane length also contribute to cell shape changes that are critical for coordinated migration (Maruyama and Andrew 2012). Since we observed defects in both SG lumen morphology and cellular rearrangements, we sought to explore whether SJ mutant SG cells have altered membrane lengths. To accomplish this, we measured the length of two lateral membranes per cell, and the intervening apical membrane, for a total of 10 cells per gland in stage 11, stage 13, and stage 16 *w^1118^*, *Mcr^1^*, and *Cont^ex956^* animals (Table 3). *Mcr^1^* and *Cont^ex956^* SG cells have apical membranes that are significantly shorter in length than in *w^1118^* SG cells in stage 11 embryos, yet are equivalent to *w^1118^* in length by stage 13. Interestingly, the apical membrane span decreases in the *Mcr^1^* mutant glands, while the apical membrane of *Cont^ex956^* and *w^1118^* glands remain relatively constant from stages 13 to 16. On the lateral membrane, we found that both *Mcr^1^* and *Cont^ex956^* mutant glands had similar lengths to *w^1118^* at stage 11 and 13 SGs, whereas *Mcr^1^* mutant SG cells had significantly longer lateral membrane length compared to *w^1118^* at stage 16. Since this phenotype was not observed in *Cont^ex956^*, we examined stage 16 SGs from two additional SJ mutants, *kune^c309^* and *Nrg^14^*, and found that only the lateral membrane length in *kune^c309^* was significantly different from *w^1118^* (Table 3). We also used *Forkhead-GAL4* to specifically express RNAi against *Mcr* and *kune* in the SG. At stage 16, *Fkh > Mcr-RNAi* recapitulated both the shorter apical membrane and longer lateral membrane found in *Mcr^1^* mutant animals at stage 16 (Table 3), and *Fkh > kune-RNAi* recapitulated the longer lateral membrane found in *kune^C309^* mutant animals. Together these results reveal that as wild-type SG cells undergo a modest reduction in apical basal length between stages 11 and 16, the SG cells in some, but not all, SJ mutant animals fail to undergo this change. Furthermore, the similarity in phenotypes between the loss of function alleles and the SG-specific RNAi suggests a tissue autonomous role for these genes in normal morphogenesis.

**Table 3:** Apical and lateral membrane lengths in wild-type and SJ mutant SG cells

Genotype	Average Length of Apical Membrane Stage 11*^a^*	Average Length of Apical Membrane Stage 13*^a^*	Average Length of Apical Membrane Stage 16*^a^*	Average Length of Lateral Membrane Stage 11*^b^*	Average Length of Lateral Membrane Stage 13*^b^*	Average Length of Lateral Membrane Stage 16*^b^*
*w^1118^*	6.6 ± 0.6	4.3 ± 0.4	4.6 ± 0.3	9.8 ± 1.3	8.3 ± 2.9	8.6 ± 0.2
*Cont^ex956^*	4.6 ± 0.7*^,^*^c^*	3.7 ± 0.6	4.5 ± 0.4	11.7 ± 1.2	11.7 ± 0.9	8.6 ± 0.3
*Mcr^1^*	3.1 ± 1.3**	4.4 ± 0.9	3.9 ± 0.5***	9.9 ± 1.3	9.6 ± 2.2	10.1 ± 0.4**
*kune^C309^*	ND	ND	4.3 ± 0.5	ND	ND	9.9 ± 0.4*
*Nrg^14^*	ND	ND	4.2 ± 0.7	ND	ND	8.1 ± 0.5
*Fkh > Mcr-RNAi*	ND	ND	3.8 ± 0.5***	ND	ND	10.0 ± 0.2***
*Fkh > Kune-RNAi*	ND	ND	3.3 ± 0.3***	ND	ND	10.6 ± 0.4***

* *P* < 0.05, ** *P* < 0.001, *** *P* < 0.0001. ND, not determined.

a Average length plus standard deviation (in μm) of apical domain of 10 SG cells per gland at the given developmental stage (*n* = 3 glands).

b Average length plus standard deviation (in μm) of lateral domain of 10 SG cells per gland at the given developmental stage (*n* = 3 glands).

c Statistics indicate difference between SJ mutant and *w^1118^* at the same stage of development.

## Discussion

The purpose of this study was to reinvestigate a representative collection of SJ genes for a developmental role during essential morphogenetic events during embryogenesis in *Drosophila*. For this study we chose nine different core SJ genes including those that encode transmembrane proteins such as Nrx-IV, Contactin, the homophilic adhesion protein Nrg, a component of the ATPase complex, a claudin, as well as the cytoplasmic SJ-organizing protein Cor. Somewhat surprisingly, we found that all nine of these genes are required for embryonic developmental events as early as germ band retraction, with highly penetrant defects in head involution and SG organogenesis. Two important implications follow from these new observations. First, a large collection of SJ proteins plays essential roles in morphogenesis throughout embryonic development. Although we were not the first to notice that some SJ genes are required for DC (Fehon *et al.* 1994; Baumgartner *et al.* 1996), this study demonstrates how prevalent the requirement for SJ genes is throughout embryonic development. Second, and more importantly, the timing of developmental events requiring SJ gene function indicates that these proteins are likely required for a function that is independent of their role in forming an occluding junction in the lateral membrane.

### SJ genes are required for embryonic morphogenesis

While examining late-stage mutant embryos by immunofluorescence, we noticed a substantial number of embryos that appeared to arrest development prior to stage 16, with some failing to complete germ band retraction (Figure 2). Because these embryos failed to secrete cuticle they likely represent the high percentage of “Empty Cuticle” embryos we observed in our cuticle analyses of SJ mutants (Figure 1 and Table 1), suggesting a role for SJ genes in early developmental events. Of those mutant embryos that progressed far enough to secrete a cuticle, the most penetrant defect we observed was in head involution (Figure 1 and Table 1). We also found clear evidence for defects in dorsal closure, although scoring this phenotype by obvious holes or scabs in cuticle preparations with their vitelline envelope intact almost certainly underestimated the penetrance of these defects. Several other genes that are required for the coordination of DC lead to subtle defects that are not obvious by cuticle preparations, but clearly play important roles. For example, mutations in *wingless* perturb the organization of the dorsal most epithelial cells and result in a slower and uncoordinated closure that nevertheless goes on to completion (Kaltschmidt *et al.* 2002). Thus, to better examine the role of SJ genes in DC it will be important to examine mutant embryos with a variety of cellular markers and by live imaging.

Since most SJ genes have been studied for their role in establishing the occluding junction, there is incomplete knowledge about the expression and localization of these proteins in earlier embryos. RNA sequencing data from the modENCODE project indicate that RNA for many SJ genes is present in 0–2 hr embryos (Graveley *et al.* 2011), suggesting that they are expressed maternally. We found that at least the eight SJ proteins we examined are expressed as early as germ band extended embryos, where they are associated with the lateral membrane (Figure 3). Although we did not examine them fully, we noticed expression of some SJ proteins in even earlier embryos raising the question of whether they could be required for earlier developmental events (S.H. and R.W., unpublished observation). Since our analysis was limited to zygotic loss of function mutations, we would have missed any earlier developmental requirements that were rescued by maternal contributions. A requirement for some SJ genes in oogenesis (Batz *et al.* 2014; R. W., unpublished observation), however, precludes our ability to examine embryos with maternal/zygotic loss of function mutations in these genes.

SJ genes are also required for the organogenesis of epithelial organs that is occurring coincident with the major developmental events of midembryogenesis. We noticed that the SGs of SJ mutant embryos appeared short and fat compared to wild-type glands at stage 16 (Figure 4). This phenotype results primarily from defects in cell rearrangements that normally occur between stages 11 and 16 (Figure 5 and Table 2), although we also found evidence for defects in cell shape changes for two of the four SJ mutants we examined (Table 3).

How might the loss of SJ gene function affect morphogenesis of the SGs? Chung and Andrew found that loss of function mutations in *Cadherin 99C* (*Cad99C*) result in glands that are longer and thinner with fewer cells around the lumen of the gland, whereas overexpression of Cad99C had the opposite effect, and was similar to what we observed with the SJ mutations (Chung and Andrew 2014). They went on to show that Cadd99C localizes to the apical domain of SG cells and promotes apical character in the cells. Laprise and colleagues showed that a subset of SJ genes acts in a redundant pathway with *yurt* to maintain apical basal polarity during midembryogenesis (Laprise *et al.* 2009). Although the authors indicated that there is no strong loss of apical/basal polarity in single SJ mutants, it is possible that loss of SJ gene function may result in a subtle defect in polarity, perhaps by playing a role in vesicle trafficking. In this regard, it is noteworthy that mutations in all of these SJ genes lead to a phenotype in which the epidermis secretes a thin and underdeveloped cuticle (Figure 1; Lamb *et al.* 1998), and tracheal cells of most SJ mutants fail to secrete Serpentine or Vermiform, two chitin deacetlyases required for tracheal tube length (Luschnig *et al.* 2006; Wang *et al.* 2006). In further support of the notion that single SJ mutations may have a subtle effect on apical/basal polarity, the convoluted tracheal phenotype found in *cor* mutant animals can be partially suppressed by loss of one copy of *crumbs* (Laprise *et al.* 2010).

### The role of SJ proteins in morphogenesis is likely independent of its role in forming an occluding junction

**Figure 6:**
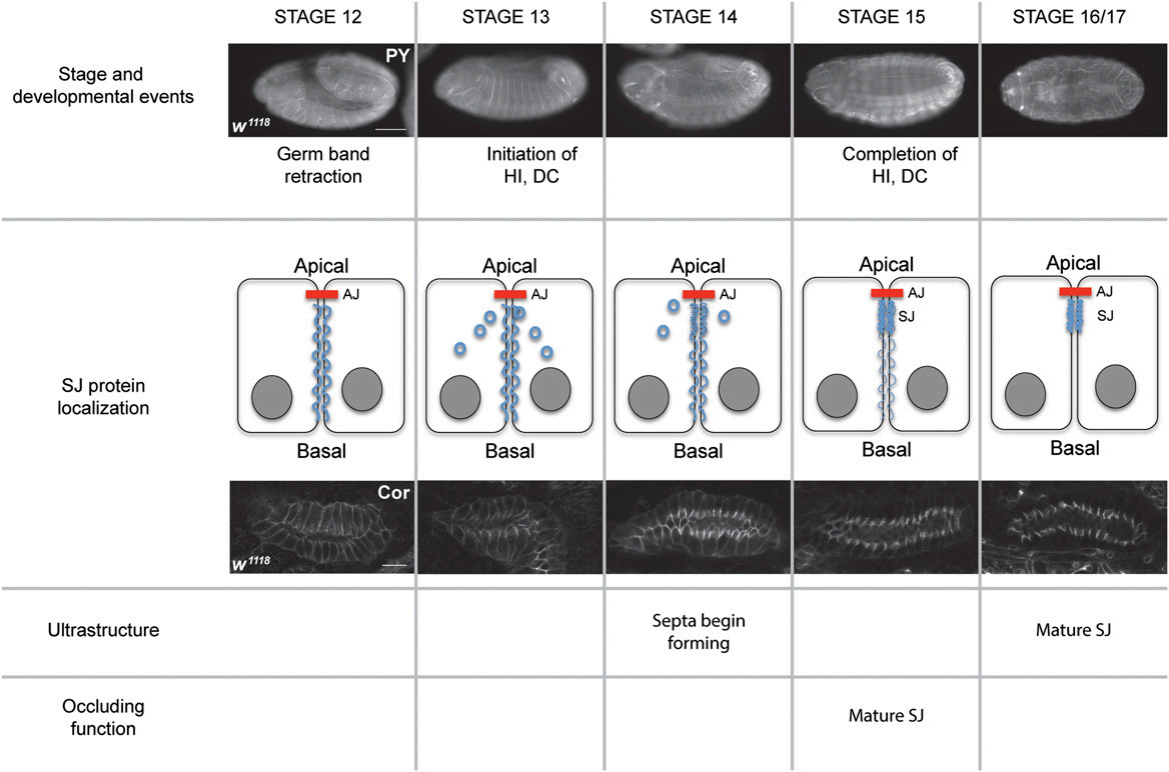
SJ biogenesis and its relationship to embryonic morphogenesis. Comparison of developmental events (top row) to SJ protein localization (second row), along with timing of SJ ultrastructure [as determined by Tepass and Hartenstein (1994)] (third row) and timing of the occluding function of the junction [as determined by Paul *et al.* (2003)] (bottom row). The top row shows fluorescence photomicrographs of *w^1118^* embryos stained with FITC-labeled antiphosphotyrosine antibodies (PY). Anterior is to the left and dorsal is up in stages 12–14 and facing in stages 15 and 16/17. Scale bar = 100 μm. Second row shows confocal optical sections of SGs from stage 12 to 16 *w^1118^* embryos stained with antibodies against Cor. Scale bar = 20 μm. Note that all major developmental events are occurring and are mostly complete before the SJ is organized and physiologically tight. See text for details. DC, dorsal closure; HI, head involution.

It is instructive to compare the timing of embryonic developmental events with the biogenesis of the SJ. Germ band retraction is complete by stage 12 of embryogenesis, whereas head involution and DC occur during stages 13 and 14 (Figure 6). Similarly, SG and hindgut morphogenesis occur between stages 11 and 15. The biogenesis of the SJ is an extended process, but the refinement of SJ proteins to the region of the SJ occurs primarily during stages 15 and 16 (Figure 6). This correlates well with ultrastructural analysis showing the formation of extracellular septa in the epidermis starting in stage 14 embryos, but not showing a mature junction until stage 17 (Tepass and Hartenstein 1994). Functionally, the SG and tracheal epithelia are not physiologically tight until late stage 15 (Paul *et al.* 2003). Together these observations suggest that a physiologically “tight” junction is not required for the major body plan morphogenetic events, nor for organogenesis in *Drosophila*. Interestingly, many SJ proteins are expressed very early in embryonic development and are only retargeted to the region of the SJ relatively late in embryogenesis (Figure 3 and Figure 6; Tiklova *et al.* 2010). We therefore propose that SJ proteins play an essential role in morphogenesis during early and midembryogenesis, and then are redeployed for a role as an occluding junction later in development. There is an interesting evolutionary implication of this model. If these functions of SJ proteins are truly independent, then the selective pressure for each function may have distinct evolutionary trajectories. A recent paper by Ganot examined the evolution of occluding junction proteins (Ganot *et al.* 2015), and concluded that the ancestral occluding junction was a septate-like junction composed of proteins closely related to the SJ proteins of *Drosophila*. At least some of these SJ genes are present in unicellular choanoflagellates (one of the last unicellular ancestors to metazoans). Core tight junction proteins, on the other hand, are not found in basal metazoan lineages, and appear to originate in chordates. As ancestral organisms with SJs evolved into chordates in which the SJ was supplanted by a tight junction, what became of the core SJ proteins? It will be interesting to identify the true orthologs of SJ genes and characterize their subcellular localization and function. We would predict that the proteins encoded by these genes will have lost their role in the occluding junction, but may still be present and function in essential morphogenetic processes in development.

## References

[bib1] Batz T. Forster D. Luschnig S., 2014 The transmembrane protein Macroglobulin complement-related is essential for septate junction formation and epithelial barrier function in *Drosophila*. Development 141: 899–908. 24496626 10.1242/dev.102160

[bib2] Baumgartner S. Littleton J. T. Broadie K. Bhat M. A. Harbecke R., 1996 A Drosophila neurexin is required for septate junction and blood-nerve barrier formation and function. Cell 87: 1059–1068. 8978610 10.1016/s0092-8674(00)81800-0

[bib3] Behr M. Riedel D. Schuh R., 2003 The claudin-like megatrachea is essential in septate junctions for the epithelial barrier function in *Drosophila*. Dev. Cell 5: 611–620. 14536062 10.1016/s1534-5807(03)00275-2

[bib45] Bradley P. L. Myat M. M. Comeaux C. A. Andrew D. J., 2003 Posterior migration of the salivary gland requires an intact visceral mesoderm and integrin function. Dev. Biol. 257: 249–262. 12729556 10.1016/s0012-1606(03)00103-9

[bib4] Buszczak M. Paterno S. Lighthouse D. Bachman J. Planck J., 2007 The Carnegie protein trap library: a versatile tool for Drosophila developmental studies. Genetics 175: 1505–1531. 17194782 10.1534/genetics.106.065961 PMC1840051

[bib5] Chung S. Andrew D. J., 2014 Cadherin 99C regulates apical expansion and cell rearrangement during epithelial tube elongation. Development 141: 1950–1960. 24718992 10.1242/dev.104166 PMC3994772

[bib6] Dietzl G. Chen D. Schnorrer F. Su K. C. Barinova Y., 2007 A genome-wide transgenic RNAi library for conditional gene inactivation in *Drosophila*. Nature 448: 151–156. 17625558 10.1038/nature05954

[bib7] Faivre-Sarrailh C. Banerjee S. Li J. Hortsch M. Laval M., 2004 *Drosophila* contactin, a homolog of vertebrate contactin, is required for septate junction organization and paracellular barrier function. Development 131: 4931–4942. 15459097 10.1242/dev.01372

[bib8] Fehon R. G. Johansen K. Rebay I. Artavanis-Tsakonas S., 1991 Complex cellular and subcellular regulation of notch expression during embryonic and imaginal development of Drosophila: implications for notch function. J. Cell Biol. 113: 657–669. 2016340 10.1083/jcb.113.3.657 PMC2288958

[bib9] Fehon R. G. Dawson I. A. Artavanis-Tsakonas S., 1994 A Drosophila homologue of membrane-skeleton protein 4.1 is associated with septate junctions and is encoded by the coracle gene. Development 120: 545–557. 8162854 10.1242/dev.120.3.545

[bib10] Ganot P. Zoccola D. Tambutte E. Voolstra C. R. Aranda M., 2015 Structural molecular components of septate junctions in cnidarians point to the origin of epithelial junctions in eukaryotes. Mol. Biol. Evol. 32: 44–62. 25246700 10.1093/molbev/msu265

[bib11] Genova J. L. Fehon R. G., 2003 Neuroglian, Gliotactin, and the Na^+^/K^+^ ATPase are essential for septate junction function in *Drosophila*. J. Cell Biol. 161: 979–989. 12782686 10.1083/jcb.200212054 PMC2172966

[bib12] Graveley B. R. Brooks A. N. Carlson J. W. Duff M. O. Landolin J. M., 2011 The developmental transcriptome of *Drosophila melanogaster*. Nature 471: 473–479. 21179090 10.1038/nature09715 PMC3075879

[bib13] Hall S. G. Bieber A. J., 1997 Mutations in the *Drosophila* neuroglian cell adhesion molecule affect motor neuron pathfinding and peripheral nervous system patterning. J. Neurobiol. 32: 325–340. 9058324

[bib14] Hall S. Bone C. Oshima K. Zhang L. McGraw M., 2014 *Macroglobulin complement*-related (Mcr) encodes a protein required for septate junction organization and paracellular barrier function in *Drosophila*. Development 141: 889–898. 24496625 10.1242/dev.102152 PMC3912832

[bib15] Izumi Y. Furuse M., 2014 Molecular organization and function of invertebrate occluding junctions. Semin. Cell Dev. Biol. 36: 186–193. 10.1016/j.semcdb.2014.09.009 25239398

[bib16] Kaltschmidt J. A. Lawrence N. Morel V. Balayo T. Fernandez B. G., 2002 Planar polarity and actin dynamics in the epidermis of *Drosophila*. Nat. Cell Biol. 4: 937–944. 12447392 10.1038/ncb882

[bib17] Lamb R. S. Ward R. E. Schweizer L. Fehon R. G., 1998 Drosophila coracle, a member of the protein 4.1 superfamily, has essential structural functions in the septate junctions and developmental functions in embryonic and adult epithelial cells. Mol. Biol. Cell 9: 3505–3519. 9843584 10.1091/mbc.9.12.3505 PMC25665

[bib18] Laprise P. Lau K. M. Harris K. P. Silva-Gagliardi N. F. Paul S. M., 2009 Yurt, Coracle, Neurexin IV and the Na^+^,K^+^-ATPase form a novel group of epithelial polarity proteins. Nature 459: 1141–1145. 19553998 10.1038/nature08067

[bib19] Laprise P. Paul S. M. Boulanger J. Robbins R. M. Beitel G. J., 2010 Epithelial polarity proteins regulate *Drosophila* tracheal tube size in parallel to the luminal matrix pathway. Curr. Biol. 20: 55–61. 20022244 10.1016/j.cub.2009.11.017 PMC2821987

[bib20] Le T. Liang Z. Patel H. Yu M. H. Sivasubramaniam G., 2006 A new family of Drosophila balancer chromosomes with a *w*^−^ *dfd*-GMR yellow fluorescent protein marker. Genetics 174: 2255–2257. 17057238 10.1534/genetics.106.063461 PMC1698648

[bib21] Llimargas M. Strigini M. Katidou M. Karagogeos D. Casanova J., 2004 Lachesin is a component of a septate junction-based mechanism that controls tube size and epithelial integrity in the *Drosophila* tracheal system. Development 131: 181–190. 14681183 10.1242/dev.00917

[bib22] Lord B. A. DiBona D. R., 1976 Role of the septate junction in the regulation of paracellular transepithelial flow. J. Cell Biol. 71: 967–972. 993276 10.1083/jcb.71.3.967 PMC2109773

[bib23] Luschnig S. Batz T. Armbruster K. Krasnow M. A., 2006 *serpentine* and *vermiform* encode matrix proteins with chitin binding and deacetylation domains that limit tracheal tube length in *Drosophila*. Curr. Biol. 16: 186–194. 16431371 10.1016/j.cub.2005.11.072

[bib24] Maruyama R. Andrew D. J., 2012 Drosophila as a model for epithelial tube formation. Dev. Dyn. 241: 119–135. 22083894 10.1002/dvdy.22775 PMC3922621

[bib25] Morin X. Daneman R. Zavortink M. Chia W., 2001 A protein trap strategy to detect GFP-tagged proteins expressed from their endogenous loci in *Drosophila*. Proc. Natl. Acad. Sci. USA 98: 15050–15055. 11742088 10.1073/pnas.261408198 PMC64981

[bib47] Myat M. M., 2005 Making tubes in the *Drosophila* embryo. Dev. Dyn. 232: 617–632. 15712279 10.1002/dvdy.20293

[bib26] Nelson K. S. Furuse M. Beitel G. J., 2010 The Drosophila Claudin Kune-kune is required for septate junction organization and tracheal tube size control. Genetics 185: 831–839. 20407131 10.1534/genetics.110.114959 PMC2907205

[bib27] Noirot-Timothee C. Smith D. S. Cayer M. L. Noirot C., 1978 Septate junctions in insects: comparison between intercellular and intramembranous structures. Tissue Cell 10: 125–136. 644571 10.1016/0040-8166(78)90011-3

[bib28] Paul S. M. Ternet M. Salvaterra P. M. Beitel G. J., 2003 The Na^+^/K^+^ ATPase is required for septate junction function and epithelial tube-size control in the *Drosophila* tracheal system. Development 130: 4963–4974. 12930776 10.1242/dev.00691

[bib29] Perrimon N., 1988 The maternal effect of *lethal(1)discs-large-1*: a recessive oncogene of *Drosophila melanogaster*. Dev. Biol. 127: 392–407. 3132409 10.1016/0012-1606(88)90326-0

[bib30] Pirraglia C. Walters J. Myat M. M., 2010 Pak1 control of E-cadherin endocytosis regulates salivary gland lumen size and shape. Development 137: 4177–4189. 21068057 10.1242/dev.048827 PMC2990209

[bib31] Schneider C. A. Rasband W. S. Eliceiri K. W., 2012 NIH Image to ImageJ: 25 years of image analysis. Nat. Methods 9: 671–675. 22930834 10.1038/nmeth.2089 PMC5554542

[bib32] Tepass U. Hartenstein V., 1994 The development of cellular junctions in the *Drosophila* embryo. Dev. Biol. 161: 563–596. 8314002 10.1006/dbio.1994.1054

[bib33] Tiklova K. Senti K. A. Wang S. Graslund A. Samakovlis C., 2010 Epithelial septate junction assembly relies on melanotransferrin iron binding and endocytosis in *Drosophila*. Nat. Cell Biol. 12: 1071–1077. 20935638 10.1038/ncb2111

[bib34] Urakabe S. Handler J. S. Orloff J., 1970 Effect of hypertonicity on permeability properties of the toad bladder. Am. J. Physiol. 218: 1179–1187. 5435418 10.1152/ajplegacy.1970.218.4.1179

[bib46] Vining M. S. Bradley P. L. Comeaux C. A. Andrew D. J., 2005 Organ positioning in *Drosophila* epithelia requires complex tissue-tissue interactions. Dev. Biol. 287: 19–34. 16171793 10.1016/j.ydbio.2005.08.017

[bib35] Wang L. Evans J. Andrews H. K. Beckstead R. B. Thummel C. S., 2008 A genetic screen identifies new regulators of steroid-triggered programmed cell death in Drosophila. Genetics 180: 269–281. 18757938 10.1534/genetics.108.092478 PMC2535680

[bib36] Wang S. Jayaram S. A. Hemphala J. Senti K. A. Tsarouhas V., 2006 Septate-junction-dependent luminal deposition of chitin deacetylases restricts tube elongation in the *Drosophila* trachea. Curr. Biol. 16: 180–185. 16431370 10.1016/j.cub.2005.11.074

[bib37] Ward R. E. Lamb R. S. Fehon R. G., 1998 A conserved functional domain of *Drosophila* coracle is required for localization at the septate junction and has membrane-organizing activity. J. Cell Biol. 140: 1463–1473. 9508778 10.1083/jcb.140.6.1463 PMC2132682

[bib38] Ward R. E. Schweizer L. Lamb R. S. Fehon R. G., 2001 The protein 4.1, ezrin, radixin, moesin (FERM) domain of Drosophila Coracle, a cytoplasmic component of the septate junction, provides functions essential for embryonic development and imaginal cell proliferation. Genetics 159: 219–228. 11560899 10.1093/genetics/159.1.219 PMC1461787

[bib39] Ward R. E. Evans J. Thummel C. S., 2003 Genetic modifier screens in Drosophila demonstrate a role for Rho1 signaling in ecdysone-triggered imaginal disc morphogenesis. Genetics 165: 1397–1415. 14668390 10.1093/genetics/165.3.1397 PMC1462826

[bib40] Wells R. E. Barry J. D. Warrington S. J. Cuhlmann S. Evans P., 2013 Control of tissue morphology by Fasciclin III-mediated intercellular adhesion. Development 140: 3858–3868. 23946443 10.1242/dev.096214 PMC3915571

[bib41] Wu V. M. Schulte J. Hirschi A. Tepass U. Beitel G. J., 2004 Sinuous is a *Drosophila* claudin required for septate junction organization and epithelial tube size control. J. Cell Biol. 164: 313–323. 14734539 10.1083/jcb.200309134 PMC2172325

[bib42] Wu V. M. Yu M. H. Paik R. Banerjee S. Liang Z., 2007 *Drosophila* Varicose, a member of a new subgroup of basolateral MAGUKs, is required for septate junctions and tracheal morphogenesis. Development 134: 999–1009. 17267446 10.1242/dev.02785 PMC1955473

[bib43] Xu N. Bagumian G. Galiano M. Myat M. M., 2011 Rho GTPase controls *Drosophila* salivary gland lumen size through regulation of the actin cytoskeleton and Moesin. Development 138: 5415–5427. 22071107 10.1242/dev.069831 PMC3222215

[bib44] Zhang L. Ward R. E., 2009 *uninflatable* encodes a novel ectodermal apical surface protein required for tracheal inflation in *Drosophila*. Dev. Biol. 336: 201–212. 19818339 10.1016/j.ydbio.2009.09.040 PMC2790384

